# Residual rates of mortality in patients with severe sepsis: a fatality or a new challenge?

**DOI:** 10.1186/2110-5820-3-27

**Published:** 2013-08-19

**Authors:** Pierre Asfar, Yann-Erick Claessens, Jacques Duranteau, Eric Kipnis, Marc Leone, Bruno Lévy, Jean-Paul Mira

**Affiliations:** 1 Service de Réanimation Médicale, University Hospital, Angers, France; 2 Urgences médico-chirurgicales, Princess Grace Hospital, Principauté de Monaco, Monaco; 3 Département d’Anesthésie-Réanimation chirurgical, University Hospital, le Kremlin-Bicêtre, France; 4 Service de Réanimation Chirurgicale, Pôle d’Anesthésie-Réanimation, Regional University Hospital, Lille, France; 5 Service d’Anesthésie-Réanimation, North Hospital, AP-HM Aix-Marseille University, Marseille, France; 6 Institut du Cœur et des Vaisseaux, Réanimation Médicale, Brabois University Hospital, Vandoeuvre-les-Nancy, France; 7 Réanimation médicale polyvalente, Cochin University Hospital, AP-HP, Paris, France; 8 Medical Intensive Care Unit, Groupe Hospitalier Cochin- Broca-Hotel Dieu 27 rue du faubourg St Jacques, 75014, Paris, France

## Abstract

Phase III clinical trials on severe sepsis and septic shock published during the past decade have failed to reveal the superiority of any therapeutic intervention on mortality compared with evolving standards of care, with the exception of the Early-Goal Directed Therapy reported in 2001. This viewpoint paper presents an analysis of these studies in order to understand what lessons can be learned and proposes perspectives for future study designs. A total of 102 studies were selected among clinical trials published in the field of severe sepsis and septic shock from 2001 to 2013, based on the assessment of a therapeutic intervention and mortality as an outcome. Studies were further selected according to randomized, controlled trial (RCT) quality criteria and analysed according to reported data. Most (n = 61) were excluded because they did not comply with RCT quality criteria or did not report inclusion criteria or patient severity (n = 22). The 19 remaining studies were categorized into three groups depending on whether the intervention assessed led to better, worse, or equivalent outcomes. It appears that the mortality rate in the control arm, ranging from 17% to 61%, impacted the results, with a benefit reported in the studies with the highest rates. Both heterogeneous studied populations and uncontrolled diversity of care among participating centres probably contributed to discrepancies between studies assessing the same intervention. The new challenge to enhance the probability of decreasing mortality rates should include a more appropriate definition of sepsis based on more specific criteria involving biomarker use and accurate patient phenotypes.

## Review

### Introduction

PROWESS-SHOCK, the most recently published trial that assessed the efficacy of activated protein C in septic shock failed to show any benefit on mortality despite an apparently appropriate design [1]. This disappointing failure, the last among many in the field of severe sepsis trials, prompted our group of intensivists particularly involved in sepsis research to look at the past decade of trials from 2001, the publication year of two positive sepsis clinical trials: the Early Goal guided therapy from Rivers and colleagues [2] and the PROWESS study [3], both published in the *New England Journal of Medicine*. In order to understand what lessons can be learned from the past, we focused on the heterogeneity of data, patient management protocols, and study populations. The interpretation is not based on a systematic review of the literature but reflects a viewpoint in the light of what we considered as the most relevant studies for the reasons explained below. Finally, this viewpoint provides the opportunity to suggest perspectives for future study designs.

### Data heterogeneity

Many randomized, controlled trials (RCTs), aiming to show an improvement of survival in patients with severe sepsis and septic shock, were performed since the initial PROWESS study and the seminal Early-Goal Directed Therapy study, both published in 2001 [2,3], in parallel with the release and widespread dissemination of the Surviving Sepsis Campaign guidelines [4].

**Figure 1:**
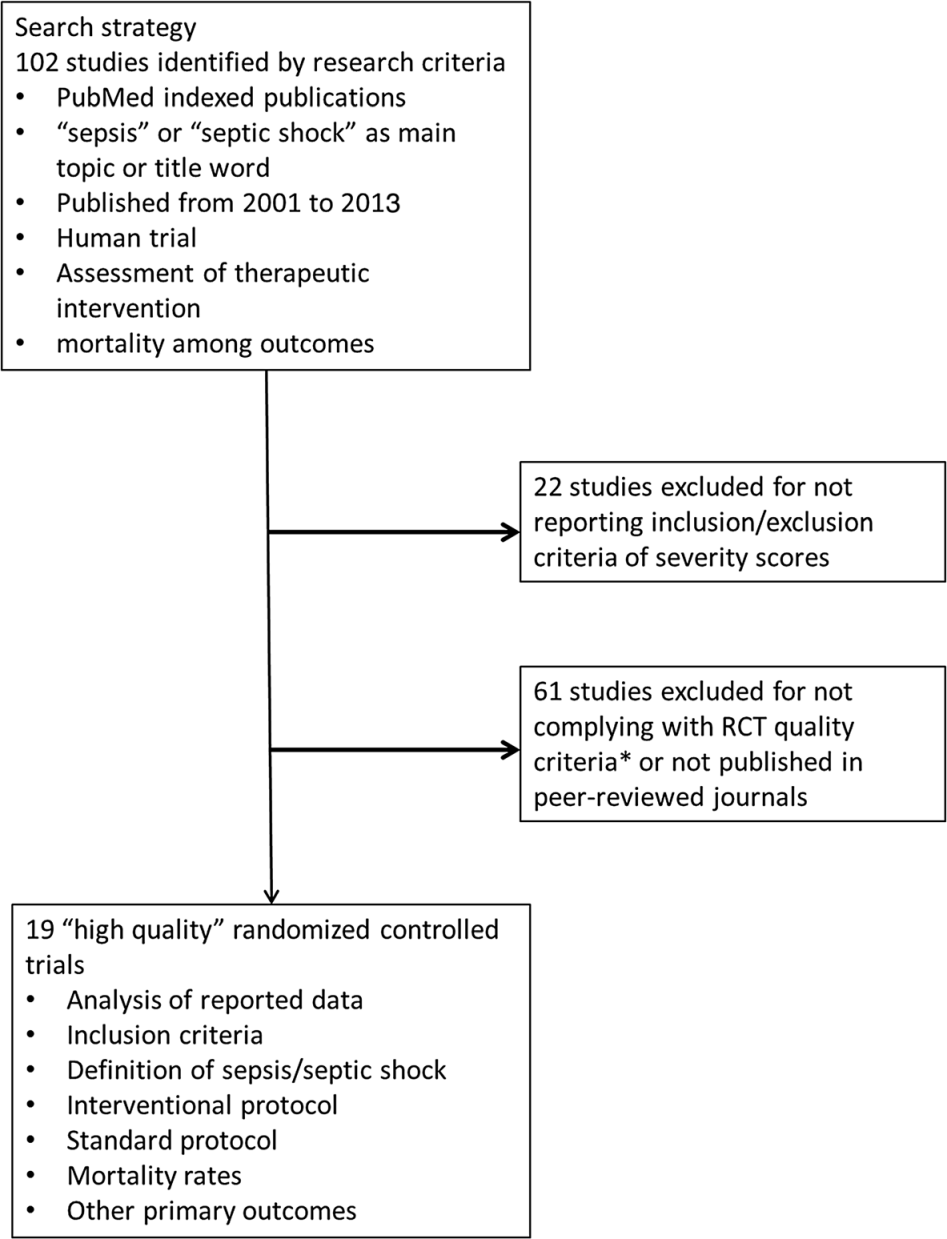
**Flow chart of study selection.** *RCT quality criteria include: appropriate and clearly focused question, randomized assignment of subjects to intervention groups, adequate concealment method, no difference between groups other than the intervention tested, all relevant outcomes measured in a valid and reliable way.

Studies that address the effect of various strategies on sepsis- or septic shock-induced mortality were selected among PubMed indexed publications from 2001 to 2013. The initial search strategy was based on “sepsis” or “septic shock” as main topics and/or title words. Studies were then qualified if they assessed at least one therapeutic intervention on mortality as an outcome. Trials that did not clearly report inclusion and exclusion criteria were excluded, as were those that did not comply with standard RCT quality criteria (e.g., appropriate research questions, randomization, blinding when relevant and inclusion/exclusion process) [5] or were not published in peer-reviewed journals [1-3,6-21]. Among 102 trials that assessed therapeutic interventions on mortality in sepsis published since 2001, only 19 were retained for further analysis (Figure 1).

The selected studies were divided into three categories regarding the impact of the studied intervention compared with the control arm: better; no difference; or worse (Table 1). Only 4 of 19 trials reported that the studied treatment successfully improved primary outcomes (mainly mortality) of patients with severe sepsis or septic shock [2,3,6,7]. In fact, most of these high-quality RCTs (15/19) found either an absence of statistical difference between the intervention and control groups (7/19) or a worse impact on at least one primary or secondary outcome (8/19) (Table 1). Hence, mortality was increased with the use of hydroxyethylstarches (HES) [21] or by L-NAME administration [16]. The increase in poor outcomes more often concerned serious adverse events: HES increased the incidence of renal failure [17,21], low-dose steroids were associated with a higher rate of new infections [18], intensive insulin therapy was reported to increase the risk of hypoglycaemia [17,20], and dopamine infusion was shown to favour arrhythmia [15].

**Table 1:** Comparison of selected randomized, controlled trials assessing the effects of therapeutic interventions on sepsis-related mortality between 2001 and 2013

**1st author and year of publication**	**Therapeutic intervention assessed (inclusion time window)**	**Mortality rate in control arm (%)**	**Impact on mortality**	**Impact on adverse events**	Effect of studied therapy*
Rivers 2001 [2]	Early goal guided therapy (As soon as possible)	49.2	Decreased mortality	No difference	Better
Bernard 2001 [3]	Drotrecogin alfa (24 h)	30.8	Decreased mortality	Serious bleeding event	
Annane 2002 [6]	Hydrocortisone + fludrocortisone (3 h)	63	Decreased mortality	No difference	
Cruz 2009 [7]	Polymyxin B hemoperfusion (6 h)	53	Decreased mortality	Cartridge clotting Hypotension and tachycardia	
Warren 2001 [8]	Antithrombin III (6 h)	38.7	No difference	No difference	No difference
Reinhart 2004 [9]	Extracorporeal endotoxin adsorber (24 h)	26		No difference	
Heinrich 2006 [10]	IG-MA enriched Ig** (not available)	28.2		Not available	
Annane 2007 [12]	Norepinephrine plus dobutamine (24 h)	34		No difference	
Russel 2008 [14]	Vasopressin (24 h)	39		No difference	
Ranieri 2012 [1]	Drotrecogin alfa (24 h)	24.2		No difference	
Opal 2013 [15]	Eritoran (12 h)	26.9		No difference	
Lopez 2004 [16]	NO synthase inhibitor LNMA (72 h)	49	Increased mortality	Low cardiac output	Worse
Abraham 2005 [11]	Drotrecogin alfa (48 h)	17	No difference	Bleeding events	
Brunkhorst 2008 [17]	Insulin/pentastarch (12 h)	26	No difference	Hypoglycemia	
				Renal failure	
				Coagulopathy	
Sprung 2008 [18]	Hydrocortisone (72 h)	31.5	No difference	Increased infections events	
Stephens 2008 [13]	G-CSF*** (24 h)	25	No difference	Higher rate of new organ failure	
Patel 2010 [19]	Dopamine Early goal guided therapy	43	No difference	Arrhythmias	
Annane 2010 [20]	Corticosteroid/Insulin (not available)	45.8	No difference	Superinfection	
				Hypoglycemia	
Perner 2012 [21]	6% HES 130/0.42 (24 h)	43	Increased mortality	Not available	

The selected trials were categorized in three groups depending on whether the effect of the studied intervention on mortality and/or serious adverse events was “better”: studied intervention leading to a significant reduction in mortality; “not different”: similar impact of the studied intervention on mortality and no severe adverse events; “worse”: studied intervention leading to a significant increase in mortality and/or to a significant increase in serious adverse events.

*Statistically significant difference *vs.* control arm: **neutropenic patients; ***granulocyte colony-stimulating factor.

It is noteworthy that two [3,7] of the four positive studies were stopped early at an interim analysis, which may impact indirect comparison between studies assessing the same intervention. For instance, the PROWESS study, that has been stopped prematurely, showed a reduction of mortality at day 28, but not at 3 months [1]. In contrast, a study on antithrombin III showed a trend towards a reduction in 90-day mortality in the high-risk SAPS II stratum, which was not observed at 28 days [8].

### Population and management heterogeneity

When comparing the selected studies, one of the most striking observations is that overall 28-day mortality rates in control groups are very heterogeneous, ranging from 17% [10,11] to 61% [6], despite similar definitions of severe sepsis and septic shock and very closed inclusion and exclusion criteria. Interestingly, three of the four positive trials are among the oldest studies and also are those with the highest mortality rates in the control group (49-61%). Conversely, negative trials included more recent trials with lower mortality rates in the control arm (17-39%) [2,3,6]. This observation was confirmed by the latest published phase III study known as the ACCESS randomized trial [15], which failed to show any benefit with the infusion of eritoran (a lipid A antagonist) on 28- or 90-day mortality, with a mortality rate in the control arm in the low range (26.9%). Another argument supporting this hypothesis is the comparison between the PROWESS [2] and PROWESS SHOCK [1] studies published 10 years apart. The authors of the PROWESS SHOCK study themselves concluded that they cannot explain the inconsistency between their findings and the reduction of mortality at 28 days that was observed in the PROWESS study. The difference in 28-day mortality rates between the two control populations was consistent (24.2% vs. 30.8%, respectively), and may reveal different severity status between the two populations/periods, and therefore explain the discrepancy in terms of benefit. Interestingly, such a difference was close to that observed between intervention and control groups in the “positive” PROWESS study.

Changes in standards of care over time, essentially based on new recommendations and guidelines for the management of sepsis, have led to a dramatic decrease in mortality rate of severe sepsis and septic shock during the past decade [22]. This fact directly impacts sepsis study designs through the necessary requirement of larger populations to adequately tailor the power of trials with mortality as a primary outcome. For instance, a 10% decrease in absolute mortality rate from a control mortality rate of 30% will require a sample size of approximately 7,000 patients (Tables 2 and 3) that has not yet been attained in critical care trials focused on sepsis.

**Table 2:** Expected change in (A) absolute mortality rates (10-60%)

**Control mortality rate (%)**	**Decreased in absolute mortality rates according to population sizes (%)**					
	**5**	**10**	**15**	**20**	**25**	**30**
10	880	0	0	0	0	0
20	1816	408	162	0	0	0
30	2490	592	250	134	82	0
40	2898	712	310	172	108	74
50	3032	762	340	192	124	86
60	2894	744	338	196	128	92

Population sizes were calculated from Stata 12.0 software with an absolute α risk of 5% (bilateral) and a power of 80%.

**Table 3:** Expected change in (B) relative mortality rates (10-90%) as a function of population sizes

**Control mortality rate (%)**	**Decreased in relative mortality rates according to population sizes (%)**								
	**10**	**20**	**30**	**40**	**50**	**60**	**70**	**80**	**90**
10	26978	6432	2720	1452	880	576	398	286	210
20	12044	2896	1236	666	408	270	190	138	104
30	7050	1714	740	404	250	168	120	88	68
40	4544	1120	490	272	172	118	84	64	50
50	3032	762	340	192	124	86	64	50	40
60	2022	522	238	138	92	66	50	40	32
70	1298	350	166	100	68	50	40	32	26
80	758	220	110	70	50	38	32	26	22
90	352	118	66	46	34	28	24	22	20

Population sizes were calculated from Stata 12.0 software with an absolute α risk of 5% (bilateral) and a power of 80%.

Additionally, virtually no information is available in these studies concerning early mortality (within the first 3 days) related to sepsis-induced refractory shock or death due to withdrawal of care in the context of persisting organ dysfunctions [23]. Such information might be important to analyse the direct effect of the tested drug and to understand the causes of death in ICU.

Another observation is that despite similar definitions of sepsis, these high-quality studies enrolled patients following varying delays from the onset of septic shock and organ failure, ranging from less than 2 h [3] to 72 h [18]. The time window ranged from “as soon as possible” to 24 h in the successful studies, whereas it ranged from 12 h to 72 h for the studies with adverse effects of therapy. Interestingly, trials that succeeded to improve outcomes were characterized by an early randomization/enrolment (within the first 24 hours) allowing early interventions. The most striking example is the Early Goal Directed Therapy trial, in which patients were enrolled within 2 hours following their arrival at the emergency department [3]. This may be a crucial point when testing drugs that aim to control the inflammatory cascade in the absence of immunomonitoring. Different delays from onset to inclusion may partially explain the different results found by two trials assessing the effects of low-dose steroids [6,18]. Indeed, patients were enrolled within 8 h in the study of Annane and colleagues reporting an improvement in the intervention arm [6], whereas enrolment was allowed within the first 72 h in the study of Sprung and colleagues reporting a potential deleterious impact of similar doses of steroids [18].

The analysis of patients’ characteristics reveals that most of the trials included heterogeneous populations. Inclusion criteria were essentially based on the 1992 ACCP consensus criteria without any further characterization of sepsis, and study groups widely varied in terms of aetiology of infection, severity of illness, organ failures, organ support, standard of care, and levels of healthcare systems. Furthermore, comorbidities, which have been shown to be major prognostic factors, were not or poorly reported in most of these studies [24]. Strikingly, stratification of patients according to severity scores (APACHE II score, SAPS…) did not prevent such bias. In a meta-analysis assessing recombinant human activated protein C and including the PROWESS and ADDRESS studies, the heterogeneity of the results (efficacy in the PROWESS study, failure in the ADDRESS study) was observed even when considering patients with APACHE II score ≥ 25 [25]. In this respect, extending the risk-related variables from the systemic inflammatory response or the organ dysfunction to a global personalized approach, such as proposed in the PIRO concept [26], might eventually contribute to a better selection of the “good patients to include”.

Overall, the analysis of the selected studies combined with the authors’ experience in the field of sepsis management supports the idea that both population heterogeneity and uncontrolled diversity of care among participating centres probably contributed to discrepancies between studies assessing the same intervention. This interpretation is consistent with the survey on the type of fluids used for fluid challenge in European ICUs [27], a study on catecholamine use [28], or the assessment of the adherence to Surviving Sepsis Campaign recommendations [29].

Finally, it is noteworthy that among the few studies reporting improved outcomes, all excepted one were academic and recruited either in a single centre [3] or in a small number of centres inside a single country [6,7]. In order to decrease the length of the trials and to favour international development of their product, pharmaceutical companies conducted large international trials, despite well-known differences in ICU bed availability and life expectancy (both indirect health system indicators) around the world. These observations support the need for improving homogeneity of populations enrolled in future trials.

## Conclusions

Although the knowledge of sepsis pathophysiology continues to dramatically progress, clinical trials in this field still suffer from major weaknesses mainly due to heterogeneity. Addressing the major causes of heterogeneity remains therefore a major issue.

What lessons have we learned from previous studies and what could be proposed to improve research in sepsis? First, “standardized” open-source clinical reporting forms should be conceived for severe sepsis/septic shock studies and should be accessible for all future studies. In an era characterized by the promises of “big-data”, this would allow massive comprehensive data aggregation into data warehouses, which could be publically available through clear usage licenses.

Second, if mortality is to remain a primary endpoint, trials should be able to recruit only patients with high mortality risks. However, the use of severity scores and classical definitions of severe sepsis/septic shock have all clearly failed to this end. Therefore, an alternative stratification of patients into high mortality risk groups by referring to the dynamic rather than the static use of existing parameters, i.e., persistence and/or worsening signs of hypoperfusion after adequate infection source control, goal-directed fluid therapy, and vasopressor infusion could be recommended. The search for panels of biomarkers associated with high mortality also could improve the selection of patients to be included into future studies. Likewise and maybe more realistically, patient phenotypes not to be included in trials because of good prognosis or absence of ongoing infection should be identified (for instance, by excluding patients with normal values of procalcitonin).

Finally, another potential cause for the heterogeneity observed in many studies seldom raised may be intercentre variability. Indeed, in many recent studies, although enrolment criteria were overall similar, the inclusion rates varied widely from centre to centre. Centres that enrol too few patients during a sustained period of time are most likely exposed to “study pitfalls”, leading to potential bias and should not be maintained in the study.

Based on our analysis highlighting the heterogeneity of data reported during the past decade in the field of severe sepsis and septic shock, the challenge is to set up new approaches, which should generate more appropriate definitions of sepsis to be used in appropriate study designs resulting in higher probabilities of showing an impact on mortality. A recent viewpoint paper [30] stated that the definition of severe sepsis vs. sepsis often is confusing and that some criteria, such as the degree of organ dysfunction, should be taken into account in the definition. One key message is that it is more appropriate to refer to various and specific sepsis instead of sepsis in general. This nuance should be taken into account in the definition and selection of patients to be enrolled.

## Appendix

The authors submitted this article on behalf of the French Opinion Group in Sepsis (FrOGS).

## Competing interests

PA has received consultancy fees and honoraria from LFB Biomédicaments. YEC has received honoraria from LFB Biomédicaments. JD has received consultancy fees from LFB Biomédicaments. EK has received consultancy fees from LFB Biomédicaments, and honoraria from LFB, Gilead, and MSD for lectures. ML has received consultancy fees from LFB Biomédicaments and honoraria from Fresenius Kabi and Novartis for lectures. BL has received consultancy fees from LFB Biomédicaments and honoraria from Pulsion and Baxter for lectures. JPM has received fees from LFB Biomédicaments, EISAI and Eli Lilly for lecture, grants or advisory boards.

## Authors’ contributions

All of the coauthors made substantial contributions to collection, analysis, and interpretation of data, were involved in drafting the manuscript and revising it critically for important intellectual content, and have given final approval of the version to be published.
